# Analysis of Biochemical and Inflammatory Markers for Predicting COVID-19 Severity: Insights From a Tertiary Healthcare Institution of Eastern India

**DOI:** 10.7759/cureus.33893

**Published:** 2023-01-17

**Authors:** Suchitra Kumari, Saurav Nayak, Swagata Tripathy, Sourin Bhuniya, Manaswini Mangaraj, Balamurugan Ramadass, Suchanda Sahu, Debapriya Bandyopadhyay, Prakruti Dash, Gautom K Saharia

**Affiliations:** 1 Biochemistry, All India Institute of Medical Sciences, Bhubaneshwar, IND; 2 Biochemistry, All India Institute of Medical Sciences, Bhubaneswar, IND; 3 Anesthesiology, All India Institute of Medical Sciences, Bhubaneswar, IND; 4 Pulmonary Medicine, All India Institute of Medical Sciences, Bhubaneswar, IND; 5 Biochemistry, All India Institute of Medical Sciences, Bhubaneswar, IOT

**Keywords:** predictive risk factors, predictive modelling, medical icu, severity scoring, covid 19

## Abstract

Background

Coronavirus disease-19 (COVID-19) patients often deteriorate rapidly based on viral infection-related inflammation and the subsequent cytokine storm. The clinical symptoms were found to be inconsistent with laboratory findings. There is a need to develop biochemical severity score to closely monitor COVID-19 patients.

Methods

This study was conducted in the department of biochemistry at All India Institute of Medical Sciences (AIIMS) Bhubaneswar in collaboration with the intensive care unit. Laboratory data of 7,395 patients diagnosed with COVID-19 during the first three waves of the pandemic were analyzed. The serum high sensitivity high-sensitivity C-reactive protein (hs-CRP, immuno-turbidity method), lactate dehydrogenase (LDH, modified Wacker et al. method), and liver enzymes (kinetic-UV method) were estimated by fully automated chemistry analyzer. Serum ferritin and interleukin-6 (IL-6) were measured by one-step immunoassay using chemiluminescence technology. Three models were used in logistic regression to check for the predictive potential of biochemical parameters, and a COVID-19 biochemical severity score was calculated using a non-linear regression algorithm.

Results

The receiver operating characteristic curve found age, urea, uric acid, CRP, ferritin, IL6, and LDH with the highest odds of predicting ICU admission for COVID-19 patients. COVID-19 biochemical severity scores higher than 0.775 were highly predictive (odds ratio of 5.925) of ICU admission (AUC=0.740, p<0.001) as compared to any other individual parameter. For the validation, 30% of the total dataset was used as testing data (n=2095) with a sensitivity of 68.3%, specificity of 74.5%, and odds ratio of 6.304.

Conclusion

Age, urea, uric acid, ferritin, IL6, LDH, and CRP-based predictive probability algorithm calculating COVID-19 severity was found to be highly predictive of ICU admission for COVID-19 patients.

## Introduction

During the third wave of the coronavirus disease-19 (COVID-19) pandemic, globally, there have been 241,886,635 confirmed cases of COVID-19, including 4,919,755 deaths [1]. The clinical manifestations were usually mild, and these patients might have had no complaint of dyspnoea, no significant increase in respiratory rate, and no respiratory distress [2]. Some people developed symptoms of severe COVID‐19 disease like shortness of breath, loss of appetite, confusion, persistent pain or pressure in the chest, and high temperature (above 38 °C). The clinical symptoms were found to be inconsistent with the severity of laboratory and imaging findings [3]. However, these patients might have deteriorated rapidly and need to be monitored closely. There is abrupt deterioration and development of complications in COVID-19 even after relatively normal initial laboratory investigations [4]. There is much concern about the mortality of COVID-19. Studies have found variation in the case fatality rate temporarily (as the epidemic has progressed) and spatially (among countries) [5]. Understanding the factors that contributed to the variation in the case fatality rate of COVID-19 would help in identifying vulnerable patients who need timely intervention [6]. This will assist the clinicians in monitoring and evaluating the severity and prognosis of COVID-19. The viral infection-related inflammation and the subsequent cytokine storm in severe cases play a crucial role in COVID-19 patient outcomes [7]. Earlier studies [8-10] have shown that severe COVID-19 patients have an elevated cytokine profile, i.e., interleukin-7 (IL-7), interleukin-8 (IL-8), interleukin-9 (IL-9), interleukin-10 (IL-10), granulocyte-macrophage colony-stimulating factor (GM-CSF), interferon-gamma (IFN-γ), fibroblast growth factor (FGF), granulocyte-colony stimulating factor (G-CSF), macrophage inflammatory protein 1 alpha (MIP1α), platelet-derived growth factor (PDGF), monocyte chemoattractant protein (MCP1), vascular endothelial growth factor (VEGF), and tumor necrosis factor (TNF-α), that is characteristic of a cytokine storm. Few recent studies proposed the association of monocyte chemoattractants, neutrophil activation signature, and pro-apoptotic factors with COVID-19 severity [11,12], and found alterations in biochemical parameters like urea, creatinine, and cystatin C (Cys C) concentrations in severe COVID-19 patients that were significantly higher than those in mild COVID-19 patients [13]. Liver biochemical parameters were found to be strongly correlated with COVID-19 mortality [14]. The results of these studies encouraged the use of clinical and laboratory parameters to develop future clinical prognosis models that would help in better understanding the pathogenesis of COVID-19. In this study, we investigated the serum high sensitive C Reactive protein (hs-CRP), ferritin, interleukin-6 (IL-6), lactate dehydrogenase (LDH), and liver enzymes in an intensive care unit (ICU) and non-ICU COVID-19 patients to determine the correlation between these parameters and the severity of COVID-19. We estimated the predictive value of these clinical laboratory parameters for the severity of COVID-19 infection.

## Materials and methods

Study population

This study was conducted in the department of biochemistry of All India Institute of Medical Sciences (AIIMS) Bhubaneswar as a retrospective comparative study. This study included laboratory data of patients diagnosed with COVID-19 and admitted to an inpatient department (IPD) and ICU of AIIMS Bhubaneswar between May 2020 and Dec 2021. The majority of the outpatients and inpatients of the institute were residents of eastern Indian states, Odisha and West Bengal. The serum high-sensitivity C-reactive protein, ferritin, IL-6, lactate dehydrogenase, urea, creatinine, uric acid, sodium, potassium, chloride, and liver function tests were the parameters performed in the clinical biochemistry laboratory of AIIMS Bhubaneswar for the COVID-19 patients. The study was approved by the Ethics Committee of AIIMS Bhubaneswar (T/IM-NF/Biochem/20/161). The biochemical reports of the COVID-19 patients at the time of admission to the hospital, without any missing parameters, were considered for analysis. The detailed demographic and clinical data of these inpatients were extracted from electronic records. All the participants with COVID-19 were divided into two groups: non-intensive care unit (non-ICU, those admitted to COVID-19 wards) and ICU (those admitted to ICU). Biochemical test results with missing serum hs-CRP, ferritin, IL-6, LDH, and liver enzymes and results beyond the linearity limit of the specific assays used were excluded. If the same patient had undergone biochemical tests multiple times during the study period, only the first result was included in the dataset.

Biochemical analysis

The serum hs-CRP, LDH, urea, creatinine, uric acid, sodium, potassium, chloride, bilirubin, and liver enzymes were estimated by a fully automated chemistry analyzer (Beckman Coulter 5800; Beckman Coulter Inc., Brea, California) using system compatible packs. Serum ferritin and IL6 were measured by Siemens Advia XP Chemiluminescence Immunoassay analyzer (Siemens Healthcare Diagnostics, Tarrytown, NEw York). CRP was estimated by the immunoturbidimetry method. Immune complexes formed in solution scattered light in proportion to their size, shape, and concentration. Turbidimeters were used to measure the reduction of incidence light due to reflection, absorption, or scatter by the immune complex formed between the CRP of the patient serum and rabbit anti-CRP-antibodies coated on latex particles. LDH was measured by a modified Wacker et al. method. Lactate and nicotinamide adenine dinucleotide (NAD+) get converted to pyruvate, and nicotinamide adenine dinucleotide (NADH) is catalyzed by LDH. NADH strongly absorbed light at 340 nm, whereas NAD did not. The rate of change of absorbance at 340 nm was directly proportional to the LDH activity in the sample. Serum ferritin was estimated by a two-site sandwich immunoassay using direct chemiluminometric technology, which used constant amounts of two anti-ferritin antibodies. IL-6 was measured by fully automated, one-step direct immunoassay using chemiluminescence technology. Liver enzymes like aspartate aminotransferase (AST), alanine aminotransferase (ALT), and alkaline phosphatase (ALP) were estimated by ultraviolet kinetic (UV-kinetic) methods by fully automated chemistry analyzer (Beckman Coulter 5800) using system compatible packs and calibrators. Quality assurance was with Bio-Rad quality control material.

After the review of the laboratory data, 7,395 COVID-19 patients' biochemical test results were selected for the current study. Of these 7,395 datasets, 70% (n=5300) were used as the training set to generate the predictive index. The rest, i.e., 2095 samples (30%), were used as the testing set.

Predictive probability calculation

Three models were used in Logistic regression to check for the predictive potential of biochemical and inflammatory parameters, and a COVID-19 biochemical severity score was calculated using a non-linear regression algorithm. COVID-19 patients were classified into high risk for ICU admission and low risk, using the following predictive probability algorithm based on non-linear regression analysis. The equation derived from the analysis was:

 -0.7+0.5 × age score+0.25 × urea score+0.15 × uric acid score+0.4 × (CRP score + ferritin score + IL6 score + LDH score) + 0.2 (if female)

Where each score is one if it is above the cut-off as determined by the ROC curve analysis of our population and parameters. A COVID-19 biochemical severity score higher than 0.775 was found to be highly predictive of ICU admission for COVID-19 patients (area under the curve (AUC)=0.740, p-value <0.001) as compared to any other individual parameter or mixed parameter. Also, it had an odds ratio of 5.925 (4.844-7.248). For the validation of the predictive probability algorithm as an efficient tool, 30% of the total dataset was used as testing data (n=2095).

Statistical analysis

All the data collected were subjected to a test of normality, following which they were expressed in terms of mean or median. As most of the parameters were biochemical, log transformation was done if, in the first instance, the data was non-parametric. The severity of the patient was determined according to the then guidelines of management laid down by the government of India and patient admission to the ICU or to the COVID-19 wards. The progression of the disease and categorization were subjected to group tests to determine any significant difference among these. A ROC was elucidated and was used to determine optimum threshold limits (cut-offs) of parameters for severity. The ROC curve analysis was done to determine how well the parameters were able to predict the ICU-admitted patients. Also, correlation between the various biochemical parameters and the severity of COVID-19 was done. Collected data from 7,395 COVID-19 patients' biochemical test results were randomly divided into a training set (70% of total data) and a test set (30% of data). Thus, 5300 COVID-19 biochemical test result data were used to formulate the COVID-19 biochemical severity algorithm, and the rest (n=2095) biochemical test results were used to test the algorithm.

## Results

In the current study, we retrospectively reviewed 7395 COVID-19 patients' biochemical test results performed in the clinical laboratory of AIIMS Bhubaneswar. The biochemical parameters of the entire study population were subjected to the Kolmogorov-Smirnov test and all the parameters were assessed to be not normally distributed; hence, nonparametric tests were used. The demographic and biochemical parameters of the entire study population were expressed as median (IQR) in Table 1.

**Table 1:** Demographic and biochemistry data of the entire population IQR - inter quartile range, mg/dL - milligram per deciliter, mEq/L - milliequivalent per deciliter, ALT/SGPT - alanine aminotransferase/ serum glutamic-pyruvic transaminase, AST/SGOT - aspartate aminotransferase/ serum glutamic-oxaloacetic transaminase, ALP - alkaline phosphatase, U/L - international units per liter, g/dL - grams per deciliter, CRP - C-reactive protein, IL-6 - interleukin 6, pg/mL - picograms per deciliter, LDH - lactate dehydrogenase

Parameter (n = 7395)		Median (IQR)
Sex	Male	4656 (62.96%)
	Female	2739 (37.04%)
Age (years)		48 (32 - 60)
Urea (mg/dl)		25 (18 - 40)
Creatinine (mg/dl)		0.9 (0.7 - 1.2)
Uric acid (mg/dl)		4.8 (3.6 - 6.3)
Sodium (mEq/L)		135 (132 - 137)
Potassium (mEq/L)		4.23 (3.89 - 4.61)
Chloride (CL-) (mEq/L)		100 (96 - 103)
Total bilirubin (mg/dl)		0.5 (0.3 - 0.7)
Direct bilirubin (mg/dl)		0.17 (0.1 - 0.3)
ALT/SGPT (U/L)		32 (19 - 57)
AST/SGOT (U/L)		36 (24 - 64)
ALP (U/L)		95 (73 - 136)
Total protein (g/dl)		7 (6.4 - 7.6)
Albumin (g/dl)		3.6 (3.1 - 4.1)
Globulin (g/dl)		3.4 (3 - 3.8)
Ionized calcium (mEq/L)		1.11 (1.01 - 1.2)
CRP (mg/L)		55.15 (14 - 138.9)
Ferritin (ng/ml)		371 (128.7 - 955.6)
IL-6 (pg/mL)		19.8 (5.9 - 60)
LDH (U/L)		300 (203 - 478)

There was a significant difference in the levels of both biochemical and inflammatory parameters, as depicted in Table 2. Urea, creatinine, uric acid, and potassium were significantly increased in ICU patients. Even the liver function tests (LFT) parameters were all significantly raised in the ICU cases. Total protein and albumin were reduced. Immunological parameters were all significantly raised. There was a marked increase in the levels of CRP, ferritin, IL-6, and LDH in ICU patients.

**Table 2:** Comparison of parameters between non-ICU and ICU patients by the Mann-Whitney U test * Chi-squared test n - group size, ICU - intensive care unit, IQR - inter quartile range, mg/dL - milligram per deciliter, mEq/L - milliequivalent per deciliter, ALT/SGPT - alanine aminotransferase/ serum glutamic-pyruvic transaminase, AST/SGOT - aspartate aminotransferase/ serum glutamic-oxaloacetic transaminase, ALP - alkaline phosphatase, U/L - international units per liter, g/dL - grams per deciliter, CRP - C-reactive protein, IL-6 - interleukin 6, pg/mL - picograms per deciliter, LDH - lactate dehydrogenase

Parameters		Non-ICU (n = 6946)	ICU (n = 449)	p-value
Sex	Male	4382 (63.09%)	274 (61.02%)	0.381*
	Female	2564 (36.91%)	175 (38.98%)	
Age (years)		47 (32 - 60)	58 (43 - 68)	<0.001
Urea (mg/dl)		25 (18 - 39)	34 (21 - 57)	<0.001
Creatinine (mg/dl)		0.9 (0.7 - 1.2)	1 (0.7 - 1.5)	<0.001
Uric acid (mg/dl)		4.8 (3.6 - 6.3)	4.9 (3.5 - 7.3)	0.007
Sodium (mEq/L)		135 (132 - 137)	135 (130 - 137)	0.144
Potassium (mEq/L)		4.22 (3.88 - 4.6)	4.335 (3.97 - 4.8)	<0.001
Chloride (CL-) (mEq/L)		100 (96 - 103)	98 (94 - 102)	<0.001
Total Bilirubin (mg/dl)		0.5 (0.3 - 0.7)	0.5 (0.3 - 0.7)	0.041
Direct bilirubin (mg/dl)		0.16 (0.1 - 0.3)	0.2 (0.1 - 0.3)	0.012
ALT/SGPT (U/L)		32 (19 - 57)	37 (22 - 60)	0.004
AST/SGOT (U/L)		36 (24 - 63)	44 (28 - 80)	<0.001
ALP (U/L)		95 (72 - 136)	101 (76 - 138)	0.069
Total protein (g/dl)		7.1 (6.4 - 7.6)	6.8 (6.2 - 7.4)	<0.001
Albumin (g/dl)		3.6 (3.1 - 4.1)	3.4 (2.9 - 3.9)	<0.001
Globulin (g/dl)		3.4 (3 - 3.8)	3.3 (2.9 - 3.7)	0.111
Ionized calcium (mEq/L)		1.11 (1.01 - 1.2)	1.12 (1.01 - 1.2)	0.670
CRP (mg/L)		47.05 (12.665 - 127.675)	108.3 (32.9 - 187.46)	<0.001
Ferritin (ng/ml)		358.9 (124.3 - 927.5)	594.5 (208.3 - 1456.9)	<0.001
IL-6 (pg/mL)		19.5 (5.4 - 57.9)	21.45 (10.5 - 71.5)	0.010
LDH (U/L)		290.05 (199 - 461)	471.5 (307 - 669.7)	<0.001

The ROC curve analysis was done to predict how well the parameters were able to predict the ICU-admitted patients (Table 3). It was seen that LDH, CRP, age, urea, and ferritin had the best predictive values among all the parameters. Apart from sodium, ALP, globulin, and ionized calcium, all other parameters were significantly able to predict ICU admission.

**Table 3:** ROC analysis with cut-off values based on Youden index n - sample size, AUC - area under curve, YI - Youden index, IQR - inter quartile range, mg/dL - milligram per deciliter, mEq/L - milliequivalent per deciliter, ALT/SGPT - alanine aminotransferase/ serum glutamic-pyruvic transaminase, AST/SGOT - aspartate aminotransferase/ serum glutamic-oxaloacetic transaminase, ALP - alkaline phosphatase, U/L - international units per liter, g/dL - grams per deciliter, CRP - C-reactive protein, IL-6 - interleukin 6, pg/mL - picograms per deciliter, LDH - lactate dehydrogenase

Parameter	n	AUC	Significance	Cut-off	YI	Sensitivity	Specificity
Age (years)	7395	0.633	<0.001	> 58	0.2123	49	72.23
Urea (mg/dl)	7139	0.616	<0.001	> 34	0.1914	49.44	69.7
Creatinine (mg/dl)	7140	0.574	<0.001	> 1	0.1563	49.66	65.96
Uric acid (mg/dl)	7127	0.538	0.014	> 6.8	0.1022	29.31	80.91
Sodium (mEq/L)	7129	0.521	0.1558	<= 130	0.05242	25.06	80.19
Potassium (mEq/L)	7112	0.558	<0.001	> 4.42	0.1055	44.82	65.73
Chloride (CL-) (mEq/L)	7130	0.556	<0.001	<= 97	0.1029	43.85	66.44
Total bilirubin (mg/dl)	7120	0.529	0.0408	<= 0.3	0.0418	30.11	74.07
Direct bilirubin (mg/dl)	7122	0.534	0.0106	> 0.29	0.06165	31.24	74.93
ALT/SGPT (U/L)	7126	0.541	0.0031	> 27	0.0943	66	43.43
AST/SGOT (U/L)	7121	0.57	<0.001	> 36	0.1254	61.74	50.79
ALP (U/L)	7127	0.526	0.605	> 101	0.5487	49.89	55.6
Total protein (g/dl)	7132	0.572	<0.001	<= 6.9	0.1269	57.72	54.97
Albumin (g/dl)	7131	0.567	<0.001	<= 3.5	0.121	57.72	54.38
Globulin (g/dl)	7128	0.522	0.1056	<= 3.4	0.04124	60.18	43.95
Ionized calcium (mEq/L)	1269	0.514	0.6684	> 1.16	0.08105	42.17	65.94
CRP (mg/L)	2715	0.649	<0.001	> 71.51	0.2178	62.65	59.13
Ferritin (ng/ml)	3753	0.604	<0.001	> 371	0.1583	64.73	51.1
IL-6 (pg/mL)	1295	0.558	0.0064	> 6.4	0.1418	85.57	28.61
LDH (U/L)	3967	0.689	<0.001	> 391	0.3028	63.64	66.64

In accordance with the cut-off scores generated by the ROC, the patients' parameters were categorized into a high or normal index. Age, urea, CRP, ferritin, IL6, and LDH had the highest odds of predicting ICU admission amongst all other parameters, as depicted in Table 4. On running a correlation by Kendall's tau-b it was seen that sodium, chloride, total protein, and albumin correlated negatively; total bilirubin, ionized calcium, and globulin showed no significance, and the rest were positively correlated.

**Table 4:** Odds ratio and correlation by Kendall's tau for parameters with ICU admission mg/dL - milligram per deciliter, mEq/L - milliequivalent per deciliter, ALT/SGPT - alanine aminotransferase/ serum glutamic-pyruvic transaminase, AST/SGOT - aspartate aminotransferase/ serum glutamic-oxaloacetic transaminase, ALP - alkaline phosphatase, U/L - international units per liter, g/dL - grams per deciliter, CRP - C-reactive protein, IL-6 - interleukin 6, pg/mL - picograms per deciliter, LDH - lactate dehydrogenase

Parameter	p-value	Odds ratio	Lower limit of 95% CI for OR	Upper limit of 95% CI for OR	Spearman's rho	p-value
Age	0.000	2.499	2.061	3.029	0.112	<0.001
Sex	0.381	1.092	0.897	1.328	0.010	0.385
Urea	0.000	2.249	1.855	2.727	0.100	<0.001
Creatine	0.000	1.912	1.578	2.317	0.079	<0.001
Uric acid	0.000	1.757	1.421	2.174	0.062	<0.001
Sodium	0.007	0.739	0.592	0.923	-0.032	0.013
Potassium	0.000	1.558	1.284	1.891	0.054	<0.001
Chloride (CL-)	0.000	0.647	0.533	0.785	-0.053	<0.001
Total bilirubin	0.052	0.813	0.659	1.002	-0.023	0.063
Direct bilirubin	0.004	1.358	1.103	1.671	0.034	0.007
ALT/SGPT	0.000	1.49	1.218	1.823	0.048	<0.001
AST/SGOT	0.000	1.666	1.369	2.028	0.061	<0.001
ALP	0.024	1.247	1.029	1.51	0.027	0.025
Total protein	0.000	0.6	0.494	0.728	-0.062	<0.001
Albumin	0.000	0.614	0.614	0.746	-0.059	<0.001
Globulin	0.089	0.844	0.694	1.026	-0.020	0.086
IC	0.133	1.411	0.898	2.218	0.042	0.151
CRP	0.000	2.427	1.956	3.011	0.158	<0.001
Ferritin	0.000	1.918	1.473	2.497	0.080	<0.001
IL-6 test	0.000	2.376	1.559	3.62	0.115	<0.001
LDH	0.000	3.496	2.682	4.557	0.155	<0.001

Three models were used in logistic regression to check for predictive potential. In model one, only inflammatory parameters were used in which CRP and LDH together could significantly predict ICU admission (AUC=0.654, p-value<0.001). In model 2, the biochemical and demographic parameters were used, and age, male gender, urea, and total protein were significant (AUC=0.663, p-value<0.001). In the third model, all parameters were used and only CRP and uric acid were significant (AUC=0.699, p-value<0.001) as shown in Table 5.

**Table 5:** Logistic regression for significant parameter model creation for predictive value for ICU admitted COVID patients Nagelkerke R2 - Nagelkerke R-squared value, AUC - area under curve, sex (1) - male, sex (2) - female, ALT - alanine aminotransferase, AST - aspartate aminotransferase, ALP - alkaline phosphatase, CRP - C-reactive protein, IL-6 - interleukin 6, LDH - lactate dehydrogenase, TBIL - total bilirubin, DBIL - direct bilirubin

Model no.	Model sample size	Model type	Model Inclusion	Model exclusion	Model significance	Nagelkerke R2	Model AUC	Model AUC sig	Significant parameter	Co-efficient	p-value
1	944	Step wise	CRP, LDH	Ferritin, IL6	<0.001	0.06959	0.654	<0.001	CRP	0.006	<0.001
									LDH	0.0003	0.0165
2	7078	Step wise	Age, sex, urea, total protein	Creatinine, uric acid, sodium, potassium, chloride, TBIL, DBIL, AST, ALPT, ALP, albumin, globulin, ionized calcium	<0.001	0.01775	0.663	<0.001	Age	0.0238	<0.001
									Sex (2)	0.2094	0.0409
									Urea	-0.0045	<0.001
									Total protein	-0.1717	<0.001
3	941	Enter	All	NA	<0.001	0.118	0.699	<0.001	CRP	0.0058	<0.001
									Uric acid	0.1042	0.0111

A 2x2 table was used to determine the sensitivity and specificity of the predictive score that was generated in both the validation and training sets of data. This showed that the predictive probability algorithm thus generated had a Sensitivity of 68.3%, specificity of 74.5%, positive predictive value of 16%, and negative predictive value of 97%. In this testing set, the odds ratio was even higher than the training set, i.e., 6.304.

## Discussion

COVID-19 represents a wide spectrum of clinical presentations, from asymptomatic to critical pneumonia, acute respiratory distress syndrome (ARDS), and even death [15]. Inflammation is a critical response mechanism resulting in the progression of COVID-19 [16]. As per the recent updates, the median time from the onset of illness to the time to experience dyspnoea was five to eight days; the median time from onset of illness to ARDS was 8-12 days; and the median time from onset of illness to ICU admission was 9.5-12 days [17]. Among all hospitalized patients, 26%-32% of patients were admitted to the ICU [18]. Therefore, the markers monitoring the progression of the disease need to be identified early for better disease outcomes.

Age and associated comorbidities are the known risk factors for COVID-19 severity. Recently published meta-analyses including 16 studies [19] have evaluated various panels of circulating biomarkers for detecting the severity of COVID-19 and reported lower levels of C-reactive protein (CRP), procalcitonin, IL-6, erythrocyte sedimentation rate (ESR), serum amyloid A, and ferritin in non-severe COVID-19 group [20]. As per the current clinical practice, radiographic findings, laboratory markers, hypoxia, and indicators of organ dysfunction predict the outcomes [21]. Significantly higher levels of lactate dehydrogenase, neutrophil-to-lymphocyte ratio, D-dimer, cardiac troponin, renal biomarkers, and lower levels of lymphocytes and platelet counts were reported in patients with severe complications of COVID-19 infection as compared to non-severe COVID-19 patients [22].

As per the current guidelines of the Centers For Disease Control And Prevention (CDC), home isolation is used to separate people infected with COVID-19 (mild, asymptomatic). Infected patients are advised to monitor symptoms and emergency warning signs like difficulty in breathing, persistent pain or pressure in the chest, and pale or blue-colored skin or lips [23]. Few investigations like CRP, procalcitonin, IL-6, ESR, ferritin, chest X-ray, and CT scan are done to assess the severity and clinical score of COVID-19 patients. The combination of these diagnostic tools does not allow the establishment of a definitive prediction of the severity of COVID-19 infection. Accurate prediction of severity and identification of high-risk patients would allow for better preparation and timely referral of patients to hospital setups or ICUs as per their needs. For all these reasons, several efforts have been made to develop practical and cost-effective risk prediction tools for COVID-19 risk estimation in patients infected with COVID-19.

An upward trend of acute-phase proteins in non-survivors and a stable or downward trend in survivors were reported in comparison studies between survivors and non-survivors [24, 25]. A recent study has demonstrated the role of presepsin (PSP) in the early diagnosis of sepsis in COVID-19 patients [26]. Presepsin was significantly higher in ICU patients and fatal cases than in non-ICU COVID-19 patients. Severe and critically ill COVID-19 patients demonstrate significant elevations of LDH, creatine kinase (CK), liver enzymes (AST and ALT), total bilirubin, blood urea nitrogen (BUN), and creatinine. Bloom et al. reported a marked increase in AST in critically ill and intubated patients [27], whereas Chen et al. found higher levels of bilirubin, AST, and γ-glutamyl transferase predominantly in fatal COVID-19 cases [28]. In the present study urea, creatinine, uric acid, and potassium were significantly increased in the ICU patients. There were significantly raised levels of total bilirubin, direct bilirubin, AST, and ALT with reduced total protein and albumin in the ICU cases, best explained as hepatic injury caused by SARS-CoV-2 infection either due to systemic inflammatory response or drug toxicity. Inflammatory parameters like CRP, ferritin, IL-6, and LDH were markedly elevated in ICU patients in the current study suggesting the fact that SARS-CoV-2 targeting the alveolar macrophages via the angiotensin-converting enzyme 2 (ACE2) receptor, might increase the cytokine secretion, i.e., IL-6 and TNF-α, which subsequently induces the elevation of various acute phase proteins. In the present study, age (>58), urea (>34), CRP (>71.5), ferritin (>371), IL-6 (>6.4), and LDH (>391) showed the highest odds of predicting ICU admission amongst all other parameters in COVID-19 patients.

Neutrophil to lymphocyte ratio (NLR) and systemic immune-inflammation index or coagulopathy screening using a disseminated intravascular coagulation (DIC) scoring system is widely used in monitoring and could be useful to predict disease severity, possible complications, and outcome of COVID-19 in-patients. Mc Rae et al. presented a biomarker, i.e., C-reactive protein (CRP), N-terminus pro B type natriuretic peptide (NT-proBNP), myoglobin (MYO), D-dimer, procalcitonin (PCT), creatine kinase-myocardial band (CK-MB), and cardiac troponin I (cTnI) based COVID-19 severity score to predict mortality in COVID-19 patients [29]. The model performance was documented in terms of area under the curve and median (interquartile range) COVID-19 severity scores of patients that died versus those that recovered using pooled estimates. In the present study, three models were used in Logistic regression to check for the predictive potential of biochemical and inflammatory parameters, and a COVID-19 biochemical severity score was calculated based on an algorithm with parameters like age, sex, urea, uric acid, ferritin, IL6, LDH, and CRP. Here parameter score is one if it is above the cut-off as determined by the ROC curve analysis of our population. A COVID-19 biochemical severity scores higher than 0.775 was found to be highly predictive of ICU admission for COVID-19 patients (AUC=0.740, p-value<0.001) as compared to any other individual parameter or mixed parameter. Also, it had an odds ratio of 5.925 (4.844-7.248). COVID-19 patients were classified into high risk for ICU admission and low risk, using the following predictive probability algorithm based on non-linear regression analysis. For the validation of the predictive probability algorithm (COVID-19 severity score) as an efficient tool, 30% of the total dataset was used as testing data (n=2095). Sensitivity of 68.3%, specificity of 74.5%, a positive predictive value of 16%, and negative predictive value of 97% with the odds ratio of 6.304, even higher than the training set was observed, suggesting the fact that the severity score would help in patient care decisions and planning for resource allocation. Timely identification of patients at risk would improve the prognosis through stabilizing measures and close monitoring.

This study has the expanded biomarker panel representing diverse processes has significantly improved the generalizability of the algorithm. There are, however, a few limitations of the study. The entire study was done on unequal groups with a large difference in sizes which may considerably be biasing the data. We followed a large number of biochemical parameters and only a few immunological ones. There may exist many other important analytes which would be affecting severity in hindsight. Also, there was no follow-up of the patients to check how their severity progressed and if there was any change in the analytical values in due course of time. Also, external dataset validation is essentially required to accentuate the clinical application of our scoring modality.

## Conclusions

Age, urea, uric acid, ferritin, IL-6, LDH, CRP, and sex are the biochemical and inflammatory parameters with predictive potential. A COVID-19 biochemical severity score higher than 0.775, calculated based on a predictive probability algorithm, was found to be highly predictive of ICU admission for COVID-19 patients as compared to any other individual parameter or mixed parameter with an odds ratio of 5.925 (4.844-7.248). External validation with another set of populations would substantiate the COVID-19 biochemical severity score and improve generalizability.
